# Safety and feasibility of umbilical cord mesenchymal stem cells in patients with COVID‐19 pneumonia: A pilot study

**DOI:** 10.1111/cpr.12947

**Published:** 2020-11-17

**Authors:** Ying Feng, Jianying Huang, Jianyuan Wu, Yan Xu, Bo Chen, Lijun Jiang, Hui Xiang, Zhiyong Peng, Xinghuan Wang

**Affiliations:** ^1^ Department of Critical Care Medicine Zhongnan Hospital of Wuhan University Wuhan China; ^2^ Wuhan Leishenshan Hospital Wuhan China; ^3^ Clinical Trial Center Zhongnan Hospital of Wuhan University Wuhan China; ^4^ Jilin Tuohua Biotechnology Co., Ltd. Changchun Jilin China

**Keywords:** coronavirus disease‐2019, cytokine storm, safety, umbilical cord mesenchymal stem cells

## Abstract

**Objectives:**

We aim to explore the safety and feasibility of umbilical cord mesenchymal stem cells (UC‐MSCs) transplantation in patients with severe and critically severe coronavirus disease‐2019 (COVID‐19).

**Methods:**

We conducted a small sample, single arm, pilot trial. In addition to standard therapy, we performed four rounds of transplantation of UC‐MSCs in sixteen patients with severe and critically severe COVID‐19. We recorded adverse events from enrolment to Day 28. We evaluated the oxygenation index, inflammatory biomarkers, radiological presentations of the disease and lymphocyte subsets count on the 7th day (D7 ± 1 day), the 14th day (D14 ± 1 day) and the 28th day (D28 ± 3 days).

**Results:**

There were no infusion‐related or allergic reactions. The oxygenation index was improved after transplantation. The mortality of enrolled patients was 6.25%, whereas the historical mortality rate was 45.4%. The level of cytokines estimated varied in the normal range, the radiological presentations (ground glass opacity) were improved and the lymphocyte count and lymphocyte subsets (CD4^+^ T cells, CD8^+^ T cells and NK cells) count showed recovery after transplantation.

**Conclusions:**

Intravenous transplantation of UC‐MSCs was safe and feasible for treatment of patients with severe and critically severe COVID‐19 pneumonia.

## Graphical abstract

We investigated the safety, feasibility and clinical efficacy of Umbilical Cord Mesenchymal Stem Cells (UC‐MSCs) transplantation in severe and critically severe COVID‐19 patients. The transplantation of UC‐MSCs was safe and feasible. The oxygenation index and radiological presentations were improved. Lymphocyte and its subset counts were recovered.

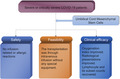

## 1 INTRODUCTION

Since the first case reported in December 2019 in Wuhan, novel coronavirus disease‐2019 (COVID‐19) has grown into a global public health emergency. The total infected number has reached 32 000 000 with more than 979 000 deaths in 216 countries, areas or territories. It is important to find a safe and effective treatment for this COVID‐19 besides controlling the pandemic. Although clinicians and researchers have tried their best to find a solution, there is no specific cure for COVID‐19 to date.

As the virus can cause a terrible cytokine storm in the lung, such as Interleukin‐2 (IL‐2), Interleukin‐6 (IL‐6), Interleukin‐7 (IL‐7), interferon‐induced protein 10 (IP10), monocyte chemoattractant protein 1 (MCP1), macrophage inflammatory protein 1α (MIP1α) and tumour necrosis factor‐α (TNF‐α), followed by oedema, dysfunction of the air exchange, acute respiratory distress syndrome (ARDS), acute cardiac injury, secondary infection, leading to sepsis and multiorgan failure, which may lead to death,^1^ any treatment that contributes to inhibiting the terrible cytokine storm will represent a major step forward.

Under this situation, stem cell therapy has become a promising therapeutic strategy due to its potential of self‐renewal, multipotent differentiation, anti‐inflammatory and immune regulatory functions. Stem cells can be attracted to the site of injury to contribute to organ repair and can foster endogenous progenitor cell function in the lung.^2^ Mesenchymal stem cells (MSCs) do not trigger a host response or cell rejection response due to their insensitivity to pro‐inflammatory interferon‐γ (IFN‐γ)‐induced human leukocyte antigen‐II (HLA‐II) expression,^3^ making them safer than other kinds of stem cells. Leng et al^4^ have reported that MSC transplantation improves the outcome of seven enrolled patients with COVID‐19 pneumonia in Beijing. After intravenous infusion, MSCs accumulate in the lung, which could improve the pulmonary microenvironment, protect alveolar epithelial cells, prevent pulmonary fibrosis and improve lung function.^5^ Meanwhile, MSCs can secrete many types of cytokines by paracrine secretion or make direct interactions with immune cells leading to immunomodulation.

Here, we conducted a pilot study to evaluate the feasibility and safety of intravenous infusion of umbilical cord MSCs (UC‐MSCs) in severe and critically severe COVID‐19 patients.

## 2 METHODS

### 2.1 Study design

A pilot trial of intravenous infusion of UC‐MSCs in sixteen severe and critically severe COVID‐19 patients was conducted in Zhongnan Hospital of Wuhan University and Wuhan Leishenshan Hospital, Wuhan, Hubei, China, and approved by the Institutional Ethics Committee of the Zhongnan Hospital of Wuhan University (Clinical Ethical Approval NO. 202002). The study was also registered in Clinical Trials (NCT 04269525).

### 2.2 Patients

**Figure 1:**
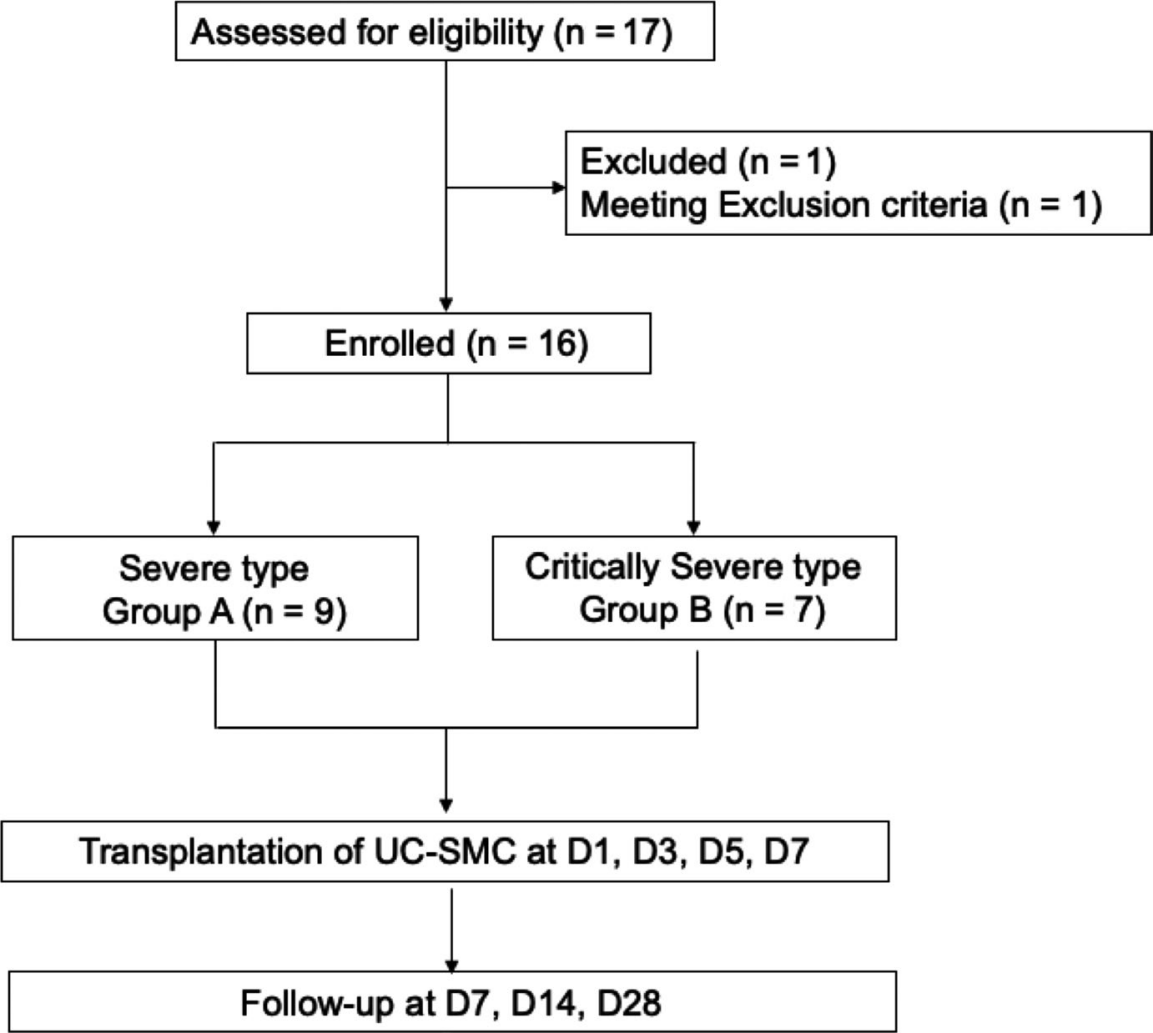
Flow diagram for patient enrolment, intervention and follow‐up

Patients were enrolled from 7 February to 1 April 2020. We invited 40 patients, only 17 (42.5%) of them or their legal representatives signed the formed consents. Sixteen patients were enrolled, and a flow diagram is shown in Figure 1. In our protocol, the follow‐up time would be 28 days in total. Due to the quarantine, the actual follow‐up time was 39 days (7‐67 days). Inclusion criteria and exclusion criteria were provided in supplementary file (Table 1).

**Table 1:** Clinical classification of the COVID‐19 released by the National Health Commission of China

Severe	Critically severe
Meet any of the followings: Respiratory distress, RR ≥ 30 min^−1^ Oxygen saturation ≤ 93% at rest state Oxygenation index ≤ 300 mm Hg, 1 mm Hg = 0.133 kPa	Meet any of the following: Respiratory failure needs mechanical ventilation Shock Combined with other organ failure, patients need ICU monitoring and treatment

### 2.3 Cell preparation and transplantation

The clinical‐grade UC‐MSCs were supplied, for free, by Jilin Tuohua Biotechnology Co., Ltd. The cell product has been certified by the National Institutes for Food and Drug Control (Report number: SH201301098, SH201301175, SH201301317, SH201500350, SH201500351, SH201500477, SH201701982, SH201701983). Before the intravenous drip, UC‐MSCs were suspended in 50 mL of normal saline, and the total number of transplanted cells was 1 × 10^8^ cells once. The patients would receive four rounds of transplantation in total, with one‐day intervals in between. The transplantation was performed about 1.5 hours with a speed of 30‐60 drops per minute.

### 2.4 Measurements

Patients were assessed for basic physical parameters, β‐human chorionic gonadotropin (for females of childbearing age), COVID‐19 clinical classification, HIV antibody at the time of enrolment and SARS‐CoV‐2 nucleic acid (nasal swab) and antibody, PaO_2_, blood routine examination, blood biochemistry, urinalysis, coagulation function, inflammatory biomarkers (white blood cell (WBC) count, lymphocyte count, IL‐2, Interleukin‐4 (IL‐4), IL‐6, Interleukin‐10 (IL‐10), TNF‐α, IFN‐γ, procalcitonin (PCT) and C reactive protein (CRP)), myocardial enzymes, lymphocyte subsets (CD4^+^ T cells, CD8^+^ T cells and natural killer (NK) cells), electrocardiograph and chest imaging (X‐ray or CT) at enrolment, on the 7th day (D7 ± 1 day), the 14th day (D14 ± 1 day) and the 28th day (D28 ± 3 days). Adverse events and concomitant medication were recorded.

### 2.5 Outcome definitions

The primary outcome was the oxygenation index on D14. The secondary outcomes were as follows: (a) mortality on D28; (b) total length of hospital stay; (c) SARS‐CoV‐2 nucleic acid or antibody assay on D7, D14 and D28; (d) radiological presentations on D7, D14 and D28; (e) inflammatory biomarkers on D7, D14 and D28; (f) lymphocyte and its subsets count on D7, D14 and D28.

### 2.6 Statistical analysis

All data were collected through EpiData and then imported into the SAS 9.4 statistical package. Figures were performed with GraphPad.v8 (GraphPad software). Categorical variables were described by frequency and proportions. Continuous variables were described by mean values when normally distributed or median and range when skewed distributed. The variables were tested using Fisher's exact or Student's t test, as appropriate. All descriptive statistical analysis was performed with SAS 9.4 (SAS Institute, Inc). All *P* values were 2‐sided, and a *P* < .05 was considered statistically significant.

## 3 RESULTS

### 3.1 Patients characteristics

A total of 16 patients with COVID‐19 were enrolled and all finished UC‐MSCs transplantation, of which nine patients were severe type and seven patients were critically severe type. The demographic information of the 16 patients is listed in Table 2. For the small sample size of patients in each type, we just used a descriptive analysis.

**Table 2:** Demographic characteristics of enrolled patients

	Severe	Critically severe	Total
N	9	7	16
Age, mean (SD), y	62.33 (11.29)	61.00 (8.94)	61.75 (10.02)
Male	6 (66.67%)	6 (85.71%)	12 (75.00%)
Comorbidities			
Hypertension	2 (22.22%)	6 (85.71%)	8 (50.00%)
Diabetes	2 (22.22%)	4 (57.14%)	6 (37.50%)
Chronic kidney failure	0	3 (42.86%)	3 (18.75%)
Hepatitis B	1 (11.11%)	0	1 (6.25%)
Bronchial asthma	1 (11.11%)	0	1 (6.25%)
Alzheimer's disease	1 (11.11%)	0	1 (6.25%)
Anaemia	1 (11.11%)	0	1 (6.25%)

### 3.2 The safety and feasibility

No acute infusion‐related or allergic reactions were observed within two hours after transplantation. Similarly, no delayed hypersensitivity or secondary infections due to UC‐MSCs transplantation were detected after treatment. There were two severe adverse events (SAE) during the trial. These two patients suffered from bacterial pneumonia and septic shock, which were lethal complications of COVID‐19. A previous report from Wuhan showed that bacterial pneumonia and septic shock occurred in 21.2% and 15% of ICU patients, respectively.^6^ The two SAEs were considered to have no relationship with UC‐MSCs transplantation. The first SAE was from a male patient, diagnosed as critical severe type COVID‐19 pneumonia with hypertension and diabetes and accepted three times transplantation of UC‐MSCs. The patient worsened on D3 due to bacterial infection with elevated white blood cell count and PCT. He developed multiorgan function failure on D5 and died on D6. The bacterial culture of UC‐MSCs was negative. The second SAE was from a female patient, diagnosed as critical severe type COVID‐19 pneumonia with Alzheimer's disease and finished four times transplantation of UC‐MSCs. The patient worsened on D13 due to aspiration. We upgraded antibiotics and enhanced airway care. However, the patient did not improve after the treatment and died on D23 due to circulation and respiratory failure. In the survived 14 patients, adverse events included hypoproteinemia, sleeplessness, gastrointestinal disease and paroxysmal arrhythmia.

### 3.3 The primary outcome

**Figure 2:**
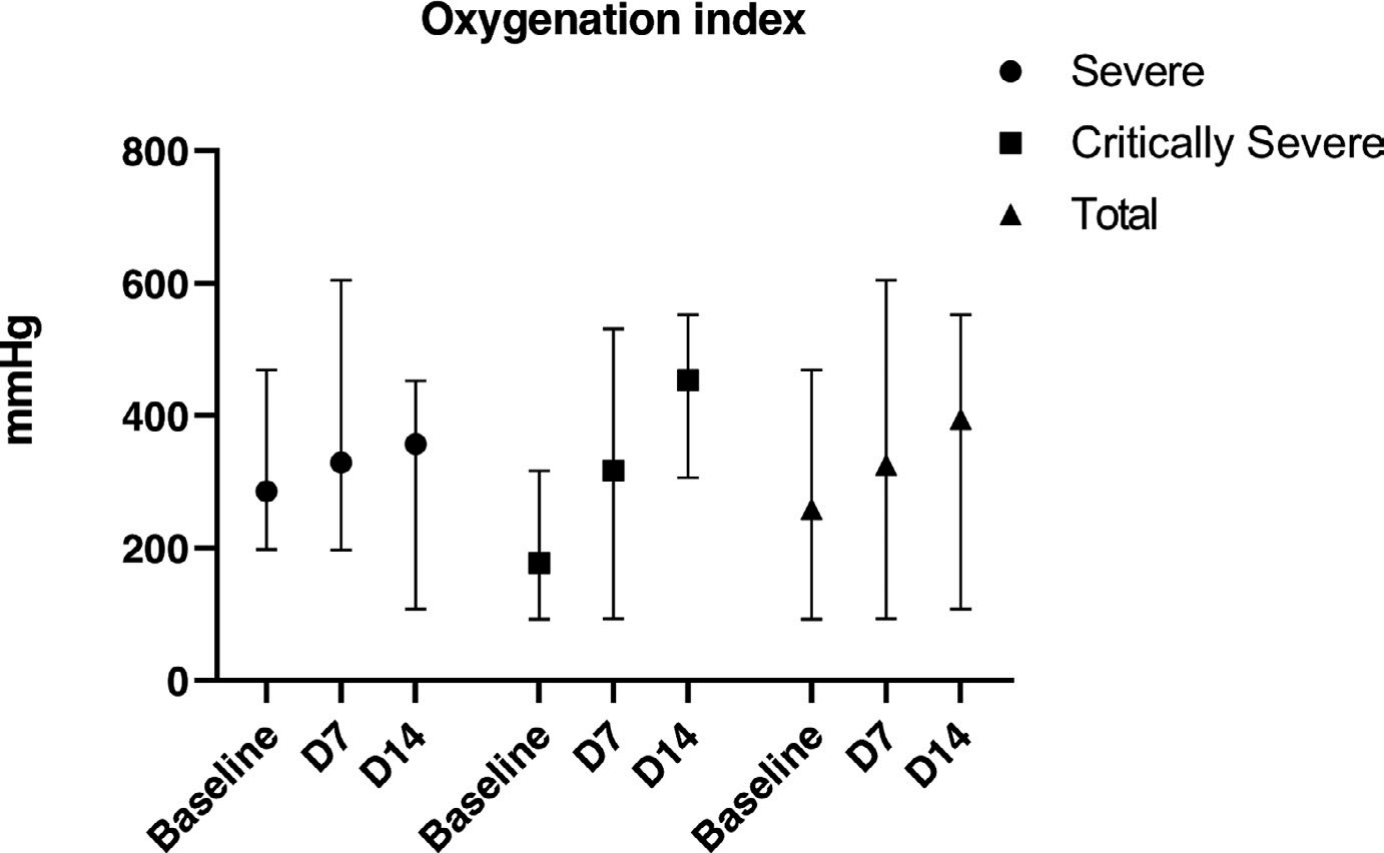
Oxygenation index at baseline (7 days before transplantation), D7 and D14 post‐treatment. Data were represented in median and range

The primary outcome was the oxygenation index on D14. Before UC‐MSCs transplantation, the oxygenation index in severe type patients (n = 8) and critically severe type patients (n = 7) was 285.50 (197.50‐469.00) mm Hg and 177.14 (92.50‐316.00) mm Hg, respectively with the mean oxygenation index in total 258.80 (92.50‐469.00) mm Hg. After four times transplantation, the oxygenation index increased into 329.00 (197.70‐604.00) mm Hg and 316.84 (93.30‐531.00) mm Hg in severe type patients (n = 9) and critically severe type patients (n = 6) with the oxygenation index in total 325.70 (93.30‐604.00) mm Hg on D7. However, 5 severe type patients and three critically severe type patients missed the arterial blood gas analysis on D14. The oxygenation index was 356.95 (107.50‐452.40) mm Hg and 453.79 (306.00‐552.30) mm Hg in severe type patients (n = 4) and critically severe type patients (n = 4) with the oxygenation index in total 394.79 (107.50‐552.30) mm Hg on D14. Despite the small number of enrolled patients, the oxygenation index was improved after UC‐MSCs transplantation (Figure 2).

### 3.4 The secondary outcome

**Figure 3:**
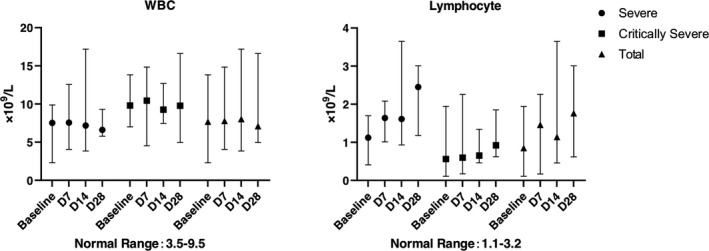
The white blood cell count and lymphocyte count at baseline (7 days before transplantation), D7, D14 and D28 post‐treatment

**Figure 4:**
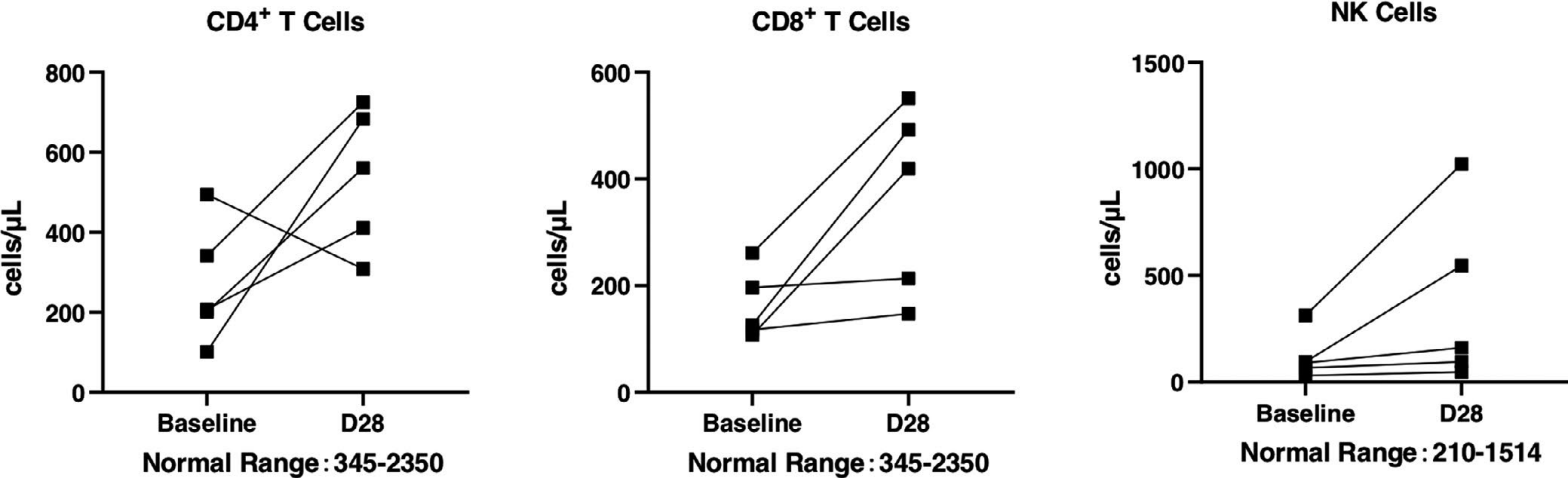
The lymphocyte subsets count at baseline (7 days before transplantation), and D28 post‐treatment

**Figure 5:**
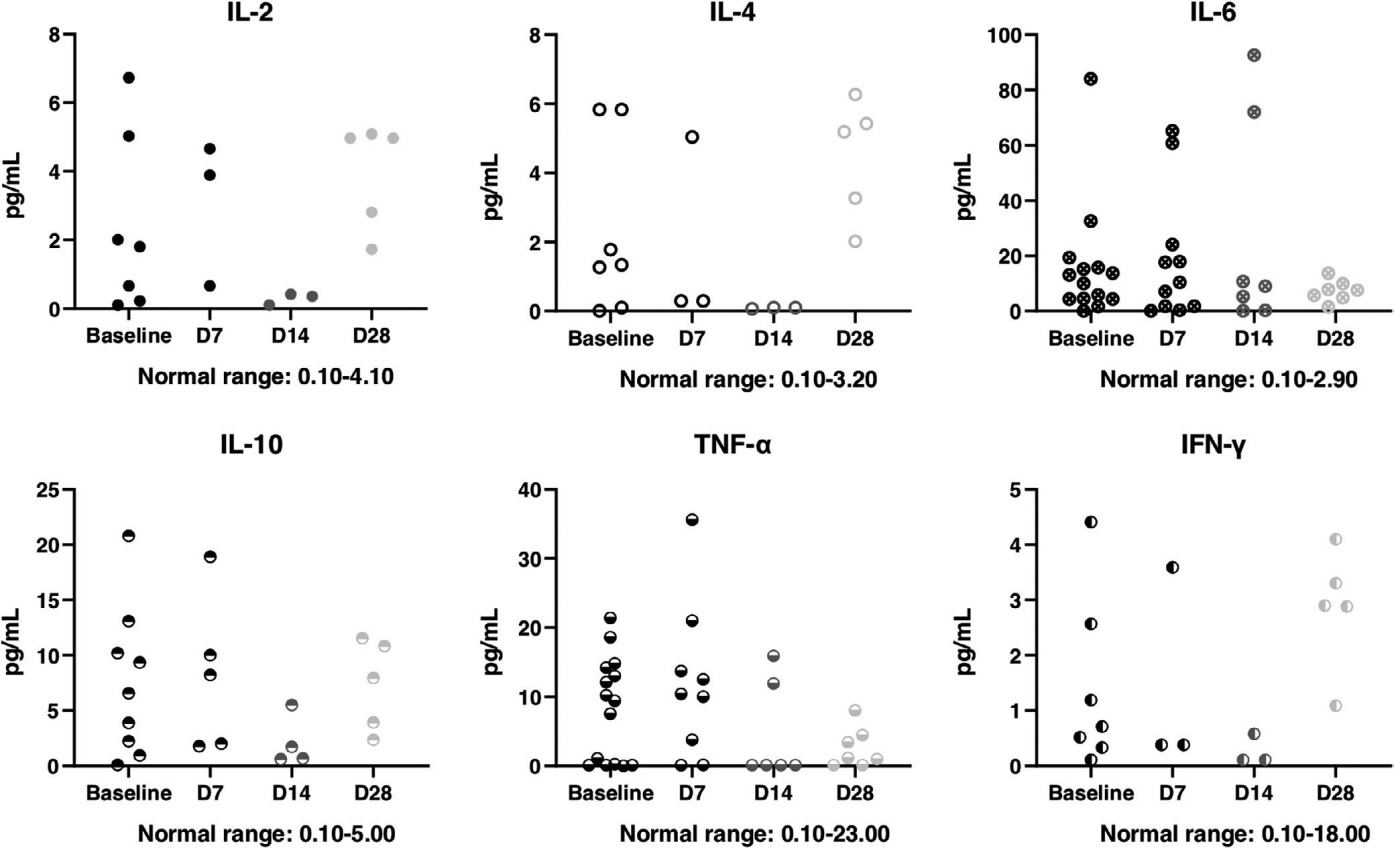
The cytokines at baseline (7 days before transplantation), D7, D14 and D28 post‐treatment

**Figure 6:**
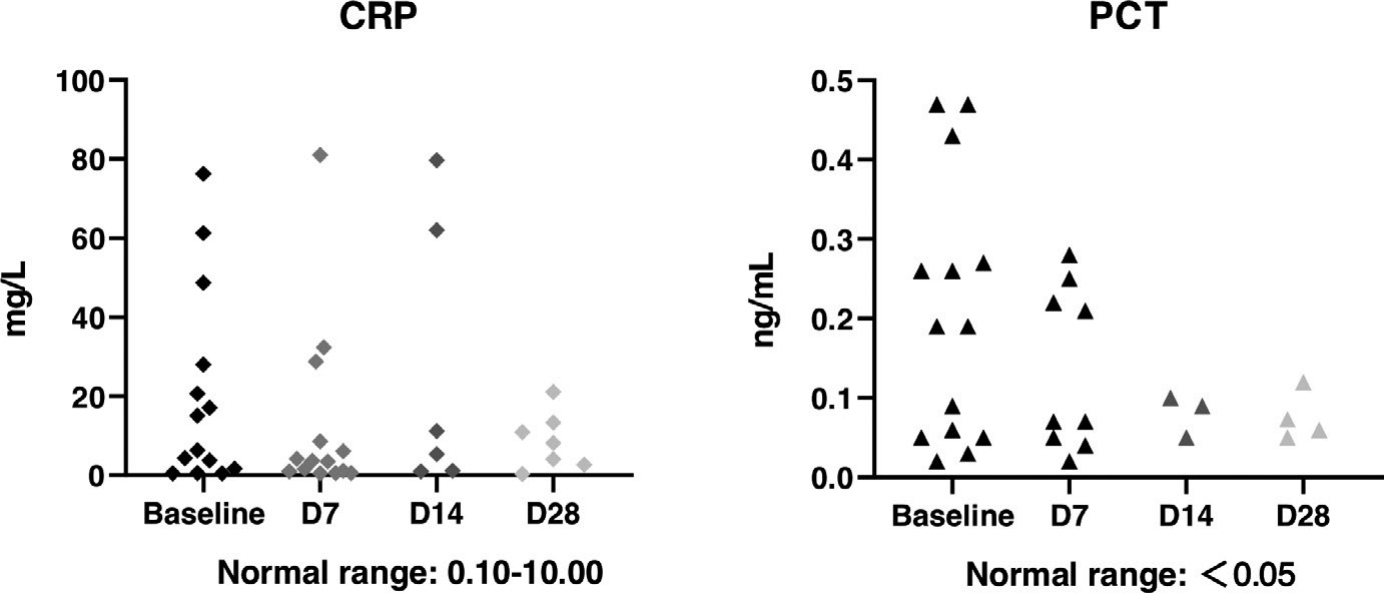
The C reaction protein (CRP) and procalcitonin (PCT) level at baseline (7 days before transplantation), D7, D14 and D28 post‐treatment

The mortality on D28 in total was 6.25%. One severe type patient died, and one critically severe type patient died. There was no statistical significance between severe type and critically severe type (*P* = 1.0000). The SARS‐CoV‐2 nucleic acid and antibody assay results were shown in Tables 3, 4. An independent radiologist reported the radiological presentations (ground glass opacity), and all showed improvement compared with baseline. The white blood cell count was similar in each follow‐up, whereas lymphocyte count showed recovery after UC‐MSCs transplantation (Figure 3). Due to the lack of lymphocyte subsets test in Leishenshan Hospital, we only got 5 patients’ results enrolled in Zhongnan Hospital of Wuhan University. The lymphocyte subsets count, including CD4^+^ T cells, CD8^+^ T cells and NK cells, showed recovery after UC‐MSCs transplantation (Figure 4). The inflammatory biomarkers were shown in Figures 5, 6. The cytokines, including IL‐2, IL‐4, IL‐6, IL‐10, TNF‐α, IFN‐γ and CRP, varied in the normal range after UC‐MSCs transplantation. PCT level was relatively low in the enrolled patients.

**Table 3:** SARS‐CoV‐2 nucleic acid assay

	Severe	Critically severe	Total
Baseline			
N (missing)	9 (0)	7 (0)	16 (0)
Positive, n (%)	1 (11.11)	2 (28.57)	3 (18.75)
D7			
N (missing)	7 (2)	5 (2)	12 (4)
Positive, n (%)	0 (0.00)	1 (20.00)	1 (8.33)
D14			
N (missing)	4 (5)	4 (3)	8 (8)
Positive, n (%)	1 (25.00)	0 (0.00)	1 (12.50)
D28			
N (missing)	5 (4)	4 (3)	9 (7)
Positive, n (%)	0 (0.00)	0 (0.00)	0 (0.00)

**Table 4:** SARS‐CoV‐2 antibody assay

		Severe	Critically severe	Total
Baseline	N (missing)	8 (1)	4 (3)	12 (4)
IgM	Positive, n (%)	6 (75.00)	2 (50.00)	8 (66.67)
IgG	Positive, n (%)	8 (100.00)	4 (100.00)	11 (100.00)
D7	N (missing)	6 (3)	3 (4)	9 (7)
IgM	Positive, n (%)	5 (83.33)	2 (66.67)	7 (77.78)
IgG	Positive, n (%)	6 (100.00)	3 (100.00)	9 (100.00)
D14	N (missing)	1 (8)	2 (5)	3 (13)
IgM	Positive, n (%)	0 (0.00)	0 (0.00)	0 (0.00)
IgG	Positive, n (%)	1 (100.00)	0 (0.00)	1 (33.33)
D28	N (missing)	4 (5)	2 (5)	6 (10)
IgM	Positive, n (%)	2 (50.00)	0 (0.00)	2 (33.33)
IgG	Positive, n (%)	4 (100.00)	1 (50.00)	5 (83.33)

## 4 DISCUSSION

We conducted a single arm, pilot trial of intravenous infusion of UC‐MSCs in sixteen severe and critically severe COVID‐19 patients, confirming its safety and feasibility, with a significant increase in oxygenation index and relatively low mortality. The improvement of radiological presentations, recovery of lymphocyte count and decrease of cytokine levels were also observed in our trial, making UC‐MSCs transplantation a promising treating strategy.

The safety of MSC transplantation has been identified in previous clinical trials treating ARDS.^7^, ^8^ In recent studies about MSC transplantation in COVID‐19 patients, there was no MSC‐related adverse event either. In our study, there was no acute infusion‐related or allergic reactions were observed, and no delayed hypersensitivity or secondary infections. As MSCs trend to accumulate in pulmonary circulation after infusion, the pulmonary embolism risk increased rapidly. However, there was no published report of MSCs transplantation associated with pulmonary embolism. In our study, none of the patients developed a thromboembolic event.

Multiple clinical trials using stem cell therapy to treat the COVID‐19 have been registered at www.clinicaltrials.gov. Two published studies showed that ACE2^‐^ MSC and exosomes derived from bone marrow MSC could improve the clinical outcome of COVID‐19 patients.^4^, ^9^ Following our findings, the use of UC‐MSCs could increase the oxygenation index of severe and critically severe COVID‐19 patients.

The outcome of COVID‐19 patients admitted to the ICU is poor. In a recent series of 1581 Italian patients in Lombardy Region with COVID‐19 ARDS admitted to ICU, the mortality was 26% and only 16% had been discharged.^10^ In another series of patients in Milan, Italy, the mortality was 23% and 31% had been discharged.^11^ In Vancouver, Canada, the mortality in ICU patients was 15.4%.^12^ Earlier in Wuhan, the mortality of severe and critical patients (with the same diagnostic criteria) was 45.4%.^13^ In our study, the mortality was about 6.25%. It is noteworthy that adults with COVID‐19 often present with a profound decrease in lymphocyte count, including CD4^+^ and CD8^+^ T‐cell subsets at the early stage of this disease.^13^, ^14^, ^15^ Qin et al^15^ also reported that severe cases of COVID‐19 were likely to have lower lymphocyte count compared with non‐severe patients. More recently, CD8^+^ T cells have been reported to be significantly decreased in peripheral blood in patients with COVID‐19.^16^ More importantly, CD8^+^ T cells ≤75 cells/μL were a reliable predictor for patients' mortality with COVID‐19.^17^ In our study, severe type patients had higher lymphocyte count than critically severe type. The patients survived had CD8^+^ T cells all over 75 cells/μL, while the non‐survival patient only had 18 cells/μL CD8^+^ T cells at baseline. After the transplantation of UC‐MSCs, the lymphocyte count, including CD4^+^ T‐cell subsets, CD8^+^ T‐cell subsets and NK cells, were increased, which suggested the immunomodulation effect of UC‐MSCs may play an important role in the COVID‐19 treatment.

In a subset of COVID‐19, patients who progress to pneumonia, respiratory failure and death by the end of the first week showed an extreme rise in inflammatory cytokines including IL2, IL7, IL10 and TNF‐α.^18^ High levels of expression of IL‐1β, IFN‐γ, IP‐10 have been detected in patients with COVID‐19.^1^ The serum levels of IL‐2R and IL‐6 in patients with COVID‐19 are positively correlated with the severity of the disease.^19^ In our study, cytokines, including IL‐2, IL‐4, IL‐6, IL‐10, IFN‐γ and TNF‐α, were tested. As we had to accept cytokine profile results within 7 days before enrolment, well before manifestation of the cytokine storm in some cases, the baseline cytokine profiles might have been lower and might not have reflected the real clinical situation at the point of treatment for some cases. MSC could inhibit the secretion of pro‐inflammatory cytokines, such as IL‐1, TNF‐α, IL‐6, Interleukin‐12 and IFN‐γ, thereby reducing the occurrence of cytokine storms.^20^, ^21^ After the transplantation of UC‐MSCs, the cytokine level varied in the normal range, which might prove the anti‐inflammatory effect of UC‐MSCs.

Our study has several limitations. First, the trial was lacked randomization, blinding, and comparison, with a small sample size, which made it difficult to evaluate the efficacy of UC‐MSCs. We aimed to conduct a following phase II study. However, the epidemic situation is under control in Wuhan, we suspend the design of phase II study. Second, there was no specific statistics on the total length of hospital stay. During this pandemic in China, the COVID‐19 patients were treated in different designated hospitals in different disease periods for treatment and quarantine. The hospital information systems differed greatly between different hospitals and made the hospital stay periods impossible to compare and calculate. Third, the loss rate of follow‐up and plan deviation, especially the loss of laboratory tests, was relatively higher than usual due to the quarantine policy and psychological distress after the disease, making it difficult to do statistical analysis. Fourth, although all patients enrolled were diagnosed as COVID‐19 pneumonia according to the guidance of the National Health Commission of China, only 11%‐28% of them are positive for SARS‐CoV2 by PCR/nuclear acid testing at baseline, which may influent the outcomes. However, due to small sample size and loss of laboratory tests, we could not do a subgroup analysis. Furthermore, randomized clinical trials are needed to provide more specific evidence.

In summary, our results indicate that UC‐MSCs can be safely administered in critically ill patients with COVID‐19 pneumonia and that administration of UC‐MSCs is associated with clinical benefit and changes in inflammatory and immune populations. UC‐MSCs have fast doubling times. They can be efficiently expanded in the lab, making it feasible to carry out randomization clinical trials.

## CONFLICT OF INTEREST

All authors declare no conflicts of interest.

## AUTHOR CONTRIBUTIONS

ZP and XW conceived and designed the study. YF and HX performed the patient enrolment and patient intervention. YF and BC performed the follow‐up. JW and BC completed statistical analysis. YX and LJ contributed umbilical cord mesenchymal stem cells’ preparation. JH performed quality control of the trial. YF and JH wrote the paper. All authors read and approved the final manuscript.

## Supporting information

Supplementary Material

## Data Availability

The data that support the findings of this study are available from the corresponding author upon reasonable request.
