# Supplementary material for: Safety and feasibility of umbilical cord mesenchymal stem cells in patients with COVID‐19 pneumonia: A pilot study
Source: Cell Prolif. 2020 Nov 17;53(12):e12947. doi: 10.1111/cpr.12947 (PMC7705911; doi:10.1111/cpr.12947)
Supplement: Supplementary file 1 — Supplementary Material [file CPR-53-e12947-s001.docx]

Inclusion criteria were the following: (1) 18-80 years of age; (2) diagnosed as severe or critically severe COVID-19 according to the guidance of the National Health Commission of China (diagnostic criteria was shown in Table 1); (3) a positive reverse-transcriptase–polymerase-chain-reaction assay (DAAN Gene Co., Ltd.) for severe acute respiratory syndrome coronavirus 2 (SARS-CoV-2) in a respiratory tract sample or a positive antibody assay (Vazyme Biotech Co., Ltd.) in blood tested by a designated diagnostic laboratory; (4) Male or nonpregnant female who was willing to use effective contraception during the trial, women of childbearing potential (*i.e.*, not post-menopausal or surgically sterilized) must have a negative serum pregnancy test before randomization; (5) A signed informed consent form. If a study patient is unable to provide informed consent due to their medical condition, the patient’s legally authorized representative may consent on behalf of the study patient, as permitted by local law and institutional Standard Operating Procedures.

Exclusion criteria were the following: (1) Sequential Organ Failure Assessment (SOFA) score for liver over 3 points; (2) positive for human immunodeficiency virus (HIV) antibody; (3) history of significant hypersensitivity or allergic reaction; (4) pregnant and lactating women; (5) malignant tumor patients; (6) patients who had pulmonary embolism; (7) participation in a clinical study within three months; (8) Unable or unwilling, in the opinion of the Investigator, to comply with the protocol.
